# The gut microbiota profile is associated with insulin action in humans

**DOI:** 10.1007/s00592-012-0410-5

**Published:** 2012-06-19

**Authors:** Matteo Serino, José Manuel Fernández-Real, Eduardo García Fuentes, Maribel Queipo-Ortuño, José María Moreno-Navarrete, Álex Sánchez, Rémy Burcelin, Francisco Tinahones

**Affiliations:** 1 Institut National de la Santé et de la Recherche Médicale (INSERM), Toulouse, France; 2 Unité Mixte de Recherche (UMR) 1048, Institut de Maladies Métaboliques et Cardiovasculaires (I2MC), Université Paul Sabatier (UPS), 31432 Toulouse Cedex 4, France; 3 Department of Diabetes Endocrinology and Nutrition, Institut d’Investigació Biomédica de Girona, and CIBER Fisiopatologia Obesidad y Nutricion (CB06/03/010), Instituto de Salud Carlos III, Girona, Spain; 4 Service of Endocrinology and Nutrition, Hospital Clinico Universitario Virgen de Victoria de Malaga and CIBEROBN (CB06/03/010), Instituto de Salud Carlos III, Madrid, Spain; 5 Statistics Department, Facultat de Biologia UB, University of Barcelona, Avda Diagonal 645, 08028 Barcelona, Spain; 6 Institut de Recerca, Hospital Universitari Vall’Hebron, Passeig Vall d’Hebron 112-119, 08035 Barcelona, Spain

**Keywords:** Gut microbiota, Obesity, Metabolic diseases, Insulin action, DGGE

## Abstract

The role of the gut microbiota in the induction of metabolic diseases has now been increasingly recognized worldwide. Indeed, a specific gut microbiota has been shown to characterize lean versus obese phenotypes both in humans and mice. We have also recently demonstrated that a precise gut microbiota is associated with the host’s responsiveness to a high-fat diet. Therefore, we hypothesized that insulin resistance in humans could also be linked to a specific gut microbiota. To this aim, microbial DNA and RNA were extracted from the appendix contents of insulin-resistant versus insulin-sensitive obese subjects, matched for body mass index and age, and analyzed by DNA- and RNA-DGGE. Microbial DNA analysis showed that the patients fully segregated according to their degree of insulin action. Conversely, microbial RNA investigation showed that some degree of homology still existed between insulin-sensitive and insulin-resistant patients. Quantitative trait analysis, ordinary least squares regression, principal components regression, partial least squares, canonical correlation analysis, and canonical correspondence analysis also showed a net separation of the two phenotypes analyzed. We conclude that a specific gut microbial profile is associated with insulin action in humans.

## Introduction

Metabolic diseases such as obesity and type 2 diabetes are characterized by alterations in energy balance which explains, at least in part, the occurrence of obesity. On one hand, the impact of genetic trait variants accounts for only up to 10 % [1] of the excessive body weight gain [2]. On the other hand, environmental factors such as stress, a sedentary lifestyle, and nutrition habit, although important, cannot explain the left-over 90 % of the pandemic progression of metabolic impairment. A recent hypothesis suggests that human beings can be considered as “super-organisms” as a result of their symbiotic association with the gut microbiota [3]. Recent data demonstrated that the profile of genes expressed by the intestinal microbiota—the gut microbiome—varies between healthy [4, 5] and even between lean and obese individuals and was considered as a specific signature of the metabolic phenotype [5]. Similarly, obese mice deleted for the leptin gene were characterized by a change in the *Bacteroidetes* to *Firmicutes* ratio, the major phyla present in the intestinal microbiota of humans [5] and mice [6]. With regard to the role of environmental factor, we and others showed the major role of a fat-enriched diet on the change in intestinal microbiota. We first identified lipopolysaccharides (LPS) from gram negative bacteria as the molecular link between gut microbiota and the chronic low-grade inflammatory tone [7, 8] induced by a high-fat diet [7, 8] that leads to insulin resistance. Hence, LPS could be considered as an initiator of metabolic diseases [9]. Altogether, gut microbiota unbalance is now considered as an important trigger of white adipose tissue (WAT) plasticity, regulating fat-storage [10], energy-harvesting [11], diet-induced obesity [12], and adiposity [13]. However, the relationship between gut microbiota and insulin action in human obesity has never been established. To this aim, we evaluated the diversity of microbial cecal (appendix) DNA and RNA in insulin-resistant versus insulin-sensitive obese subjects.

## Results

### Gut DNA microbial profiles identify clusters of patients according to insulin action

**Fig. 1:**
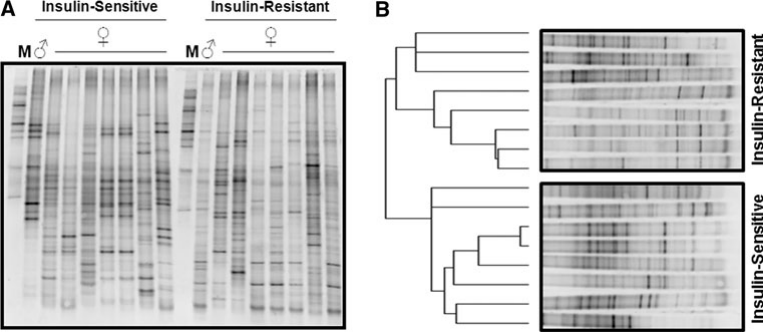
DNA gut microbial profile from the ceca of insulin-sensitive and insulin-resistant obese patients. Total DNA from both luminal and mucosal cecum (appendix) was extracted from obese insulin-resistant and obese insulin-sensitive patients. The 16S rDNA was amplified, and the amplicons were separated by electrophoresis on a gel with a denaturating gradient (DGGE). Each band was referred to as a microbial marker. The figure shows **a** the DNA-DGGE gel, with an internal marker (*M*) for electrophoresis control and **b** the cluster analysis showing the Pearson’s evolutionary tree (*left*-*side*)

**Fig. 2:**
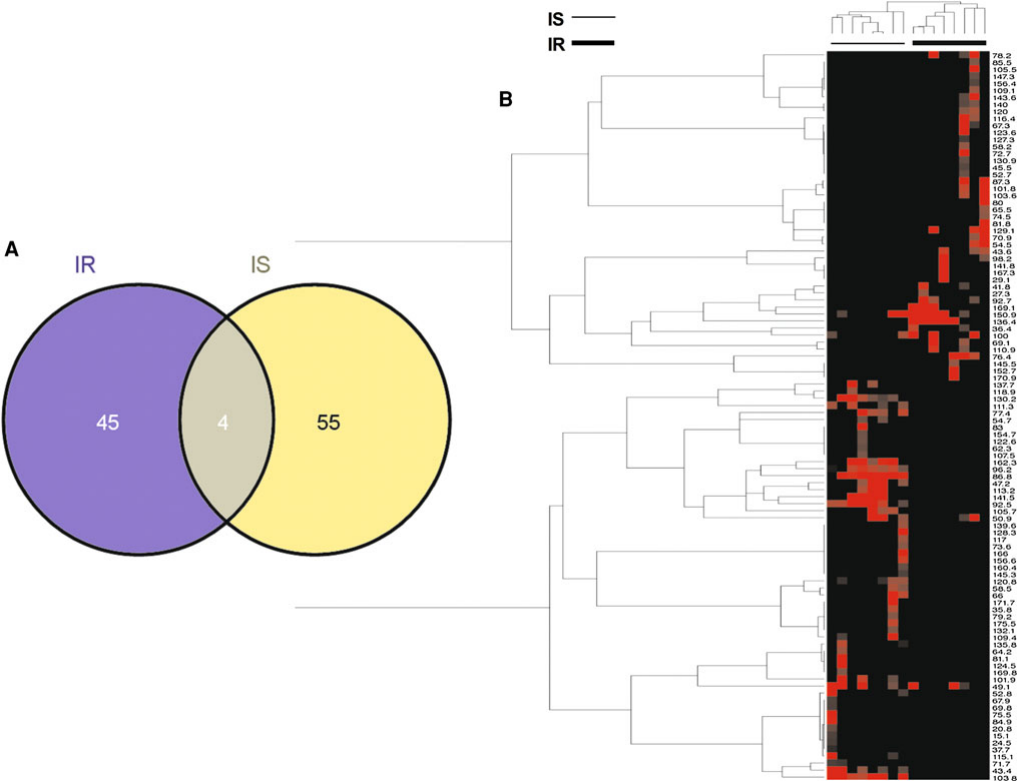
Comparative analysis of microbial markers from the DNA gut microbial profile of insulin-sensitive and insulin-resistant obese patients. **a** Venn’s diagram comparing DNA gut microbial markers and **b** heat-map based on microbial marker intensity. Pearson’s tree evolutionary analysis has been conducted with regard to marker segregation according to the clinical phenotype (IR vs. IS, *top* and *left*-*side* of the heat-map)

To determine whether a DNA-based gut microbial profile may be associated with insulin action, we performed a DNA-DGGE on the cecum (appendix) contents from 8 insulin-resistant (IR) and 8 insulin-sensitive (IS) subjects (all obese; 7 women and 1 man in both groups) comparable in age and body mass index (BMI). Notably, DNA-DGGE profiles (Fig. 1a) fully segregated according to insulin-sensitive (IS) or insulin-resistant (IR) phenotypes, as shown in Fig. 1b by the evolutionary analysis based on Pearson’s tree method. Intra-group homology was higher than inter-group homology. In detail, gut microbial profiles from both groups of patients were characterized by a specific pattern of electrophoresis bands (Fig. 1a), hereafter referred to as *microbial markers*. In fact, the IR versus IS phenotypes shared only 4 microbial markers (8 and 7 % out of the total for IR and IS, respectively), as shown by the Venn’s diagram in Fig. 2a. Therefore, 92 and 93 % of the DNA-based gut microbial profile was specific for the IR and IS phenotypes, respectively.

Next, the intensity of each microbial marker was converted into a heat-map. A Pearson’s tree evolutionary analysis allowed clusters of microbial markers to be identified which specifically belonged to a given phenotype (Fig. 2b, left-side).

We further used the Quantitative Trait Analysis, the Ordinary Least Squares regression (OLS), Principal Components Regression (PCR), Partial Least Squares (PLS), Canonical Correlation Analysis (CCA), and Canonical Correspondence Analysis (CAnoCO). Characteristic markers were obtained by selecting those variables retained in regression models such as OLS, PLS, and PCR or by selecting those variables that appeared associated with differences in IR versus IS when representing the data in reduced dimension after CCA or CANOCO. For OLS, 4 variables were retained after stepwise selection. PCR and PLS were applied without previous selection of variables, and 4 variables were retained by PCR and 9 by PLS. CCA and CANOCO were performed without any previous variable selection. Ten variables were retained by CCA and CANOCO. Combining all these methods, we found 3 DNA microbial markers (86.8 and 103.8 from IS patients; 136.4 from IR patients; numbers refer to the relative migration within the electrophoresis gel) highlighted by all methods, showing an association with insulin resistance (HOMA value) (Table 1). Moreover, 3 additional DNA microbial markers (43.4, 92.5, 96.2, all from IS patients) were highlighted by at least three approaches (Table 1).

**Table 1:** Associative analysis between DNA microbial markers and insulin resistance

Microbial marker	Correlations	IR related (Wilcox)	IR related (OLS)	Homa related (PCA-reg)	PIS	CCA	# of highlights
43.4	1	X				X	3
47.2		X					1
50.9	1						1
67.3				X	X		2
76.4						X	1
77.4		X					1
86.8	4	X	X	X	X	X	6
92.5	1	X				X	3
92.7						X	1
96.2	1	X				X	3
100	2						1
103.8	7	X	X	X	X	X	6
111.3		X	X				2
120.8		X					1
130.2	3						1
130.9		X					1
136.4	5	X	X	X	X	X	6
141.5		X					1
150.9	3					X	2
162.3		X					1
170.9						X	1

### Gut RNA microbial profile varies in accordance with insulin action

**Fig. 3:**
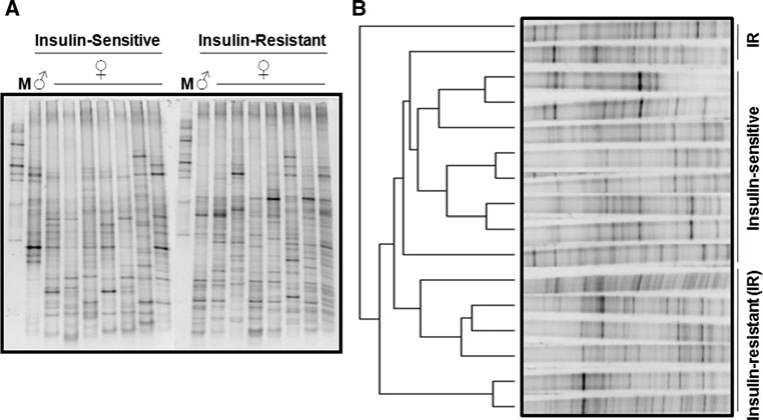
RNA gut microbial profile from ceca of insulin-sensitive and insulin-resistant obese patients. Total RNA from both luminal and mucosal cecum (appendix) was extracted from obese insulin-resistant and obese insulin-sensitive patients. The 16S rRNA was retrotranscripted, then cDNA amplified, and the amplicons were separated by electrophoresis on a gel with a denaturating gradient (DGGE). Each band was referred to as a microbial marker. The figure shows **a** the RNA-DGGE gel, with an internal marker (*M*) for electrophoresis control and **b** the cluster analysis showing the Pearson’s evolutionary tree (*left*-*side*)

To investigate whether the high level (greater than 91 %) of segregation of gut DNA microbial profiles observed in IR versus IS patients may be associated with a differential encoding activity of gut microbes, we performed a RNA-DGGE on the same samples analyzed above. Conversely to what observed on DNA-DGGE, RNA-DGGE gut microbial profiles (Fig. 3a) still showed a certain degree of homology between the IR versus IS patients. In fact, the evolutionary analysis based on Pearson’s tree method showed a subgroup of patient according to the IR or IS phenotype (Fig. 3b). Interestingly, IS patients were characterized by a higher degree of homology than IR (Fig. 3b, left-side).

**Fig. 4:**
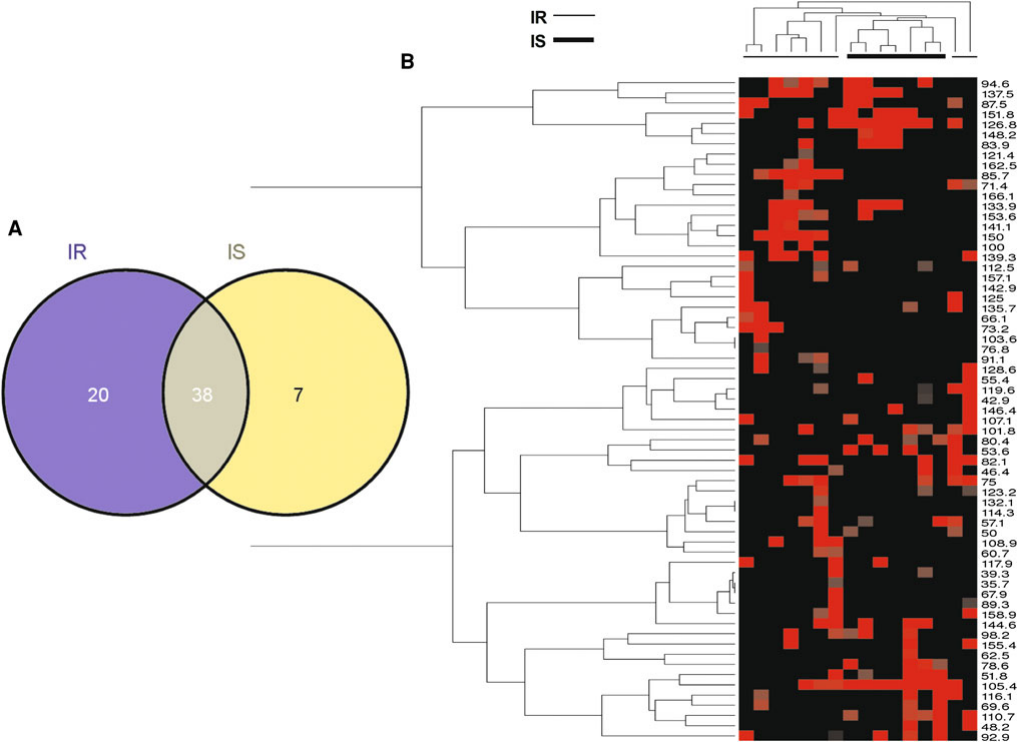
Comparative analysis of microbial markers from the RNA gut microbial profile of insulin-sensitive and insulin-resistant obese patients. **a** Venn’s diagram comparing RNA gut microbial markers and **b** heat-map based on microbial marker intensity. Pearson’s tree evolutionary analysis has been conducted with regard to marker segregation according to the clinical phenotype (IR vs. IS, *top* and *left*-*side* of the heat-map)

In detail, the IR versus IS phenotypes shared 38 microbial markers (65 and 84 % out of the total for IR and IS, respectively), as shown by the Venn’s diagram reported in Fig. 4a. Therefore, 35 and 16 % of the RNA-based gut microbial profile was specific for IR and IS phenotypes, respectively. In accordance with this result, the heat-map based on RNA microbial markers and the corresponding Pearson’s evolutionary analysis (Fig. 4b, left-side) showed clusters of segregation to a lesser extent than DNA-DGGE analysis.

## Discussion

We here report for the first time that insulin action in humans fully segregates with gut microbiota issued from the intestine, a result not yet reported from feces. This original observation suggests first a regulatory role of the microbiota which is directly in contact with intestinal cells and hence the rest of the body and second that biomarkers for the diagnosis of insulin resistance could be identified from intestinal bacterial DNA.

The role of intestinal microbiota could be due to a mechanism called bacterial translocation. It corresponds to the passage of gut indigenous bacteria through the intestinal mucosa to mesenteric lymph nodes and, in human appendix, was found to be instrumental for tolerance induction towards indigenous flora and the stimulation and normal development of the gastrointestinal-associated lymphoid tissue [14]. Substantial amounts of immune tissue associated with the appendix strongly suggest immune function capability from this gut portion.

The association of cecal appendix with substantial amount of immune tissue was considered as an indicator that the appendix may have some immune function. Recently, an improved understanding of the interactions between the normal gut flora and the immune system has led to the identification of the appendix as an apparent safe-house for normal gut bacteria. In fact, cladistic analyses, indicating that the appendix has evolved independently at least twice (once in diprotodont marsupials and once in Euarchontoglires), show a highly significant (*P* < 0.0001) phylogenetic signal in its distribution, and has been maintained in mammalian evolution for 80 millions years or longer [15]. To present, studies on gut microbiota are mostly based on fecal matter analysis [5, 16, 17]. Conversely, the aforementioned arguments strongly suggest the importance to focus on appendix microbiota as a novel actor capable to modulate host metabolism via shaping appendix immune function.

We show that a core microbiome based on gene, rather than taxon level, can be correlated with indexes of insulin resistance. Our results are in accordance with the concept of core microbiome identified in twin’s studies [16], introducing the notion that different phenotypes, that are lean versus obese, may display different patterns of gut microbes lineages nonetheless sharing a core of functions. However, it is noteworthy that further analyses based on *Omics* techniques [18] may allow to name the gut microbes associated with insulin resistance, providing a gut microbial signature for this phenotype, to be associated with the bacterial signature of obesity, already recognized in the increased *Firmicutes* to *Bacteroidetes* ratio, both in humans [5] and in mice [6].

This new concept certainly draws the route for new discoveries regarding markers of clinical phenotypes and regulatory factors of host metabolism. We recently showed that bacterial DNA, mostly issued from the *Proteobacteria* phylum, was found in the human blood. Importantly, the amount of 16S rDNA was considered as a predictive marker of the patients intended to become type 2 diabetic 6–9 years later [19]. Although the importance of the gut microbiome for host health is now widely recognized [20–22], it is not yet known which of the many hundreds of species are key for host health, and little is understood about the molecular host–microbiome interactions that influence host metabolic pathways. Each person’s gut microbial community varies in the specific bacterial lineages, with a comparable degree of co-variation between adult monozygotic and dizygotic twin pairs [16]. The origin of such variation is unknown but could be related to a given phenotypic trait [23]. In fact, a specific gut microbial profile may be responsible for a given phenotype and hence represent a target for the development of new therapy, based on the developing concept of personalized medicine [3, 24].

With that regard, we have recently showed that a specific gut microbiota is associated with a given metabolic phenotype during the phenomenon of metabolic adaptation to a high-fat diet (HFD) in mice [25]. In fact, by pyrosequencing the gut microbes issued from mice fed the same HFD and having the same genetic background, we have shown that a different *Firmicutes* to *Bacteroidetes* ratio signs the lean diabetic-sensitive versus lean diabetic-resistant metabolic phenotypes.

Therefore, our results, together with the herein presented study, corroborate the hypothesis that gut microbiota may address host responsiveness towards a given phenotype and that deviations from a core microbiome may lead to a different patho-physiologic status, that is, insulin sensitivity versus insulin resistance. A previous discovery from our laboratory showed that the blood concentration of LPS was increased in patient feeding a fat-enriched diet, whereas no difference was observed in those feeding on a carbohydrate or protein-rich diet [26]. However, no correlation was made with obesity suggesting that LPS cannot be considered as biomarker of obesity. Therefore, other factors need to be identified. In fact, insulin resistance has been found to be linked to several antimicrobial proteins that sense LPS in human plasma [27, 28], suggesting that the immune system builds specific barriers that shape our microbiota and metabolic efficiency simultaneously [29, 30].

In conclusion, we here demonstrate that intestinal bacterial DNA is a signature of insulin action in humans. Whether it has a role in the triggering or regulation of insulin resistance still needs to be determined.

## Research design and methods

Cecum intestine (appendix) from 8 insulin-resistant obese subjects and 8 insulin-sensitive obese subjects (7 women and 1 man in both groups) comparable in age and BMI (Table 2) were obtained from visceral depots during elective surgical procedures (cholecystectomy, surgery for abdominal hernia, and gastric bypass surgery). The samples were washed, fragmented, and snap-frozen in liquid nitrogen before being stored at −80º C. The subjects were invited to participate at the Endocrinology Service of the Hospital Virgen de la Victoria de Málaga (Málaga, Spain). All subjects were of Caucasian origin with no systemic disease other than type 2 diabetes or obesity, and all were infection-free during the previous month before the study. Liver disease and thyroid dysfunction were specifically excluded by biochemical work-up. Other exclusion criteria for the patients included the following: (1) clinically significant hepatic, neurological, or other major systemic disease, including malignancy; (2) history or current clinical evidence of hemochromatosis; (3) history of drug or alcohol abuse, defined as > 80 g/day, or serum transaminase activity more than twice the upper limit of normal; (4) an elevated serum creatinine concentration; (5) an acute major cardiovascular event in the previous 6 months; (6) acute illnesses and current evidence of acute or chronic inflammatory or infective diseases; and (7) mental illness rendering the subjects unable to understand the nature, scope, and possible consequences of the study. All subjects gave written informed consent after the purpose of the study was explained to them. The local board of the Hospital and the Ethics Committee approved the protocol.

**Table 2:** Anthropometrical and biochemical variables of subjects in the study

	Insulin-sensitive subjects	Insulin-resistant subjects	*P* value
*N*	7 women/1 man	7 women/1 man	
Age (years)	46.28 ± 12.64	43.12 ± 8.79	0.5
BMI (kg/m^2^)	55.8 ± 6.2	54.42 ± 5.2	0.6
WHR	0.87 ± 0.06	0.85 ± 0.08	0.6
SBP (mmHg)	141 6 ± 21.7	141 ± 15.9	0.9
DBP (mmHg)	87.1 ± 7.4	80 ± 7.9	0.15
Total cholesterol (mg/dl)	214.6 ± 40.8	208.7 ± 19.79	0.7
HDL cholesterol (mg/dl)	46.6 ± 9.7	50.42 ± 14.5	0.5
LDL cholesterol (mg/dl)	137.9 ± 23.4	126.6 ± 19.2	0.3
Fasting triglycerides (mg/dl)	94.12 ± 27.6	176.5 ± 82.2	0.02
Free fatty acids (mmol/l)	0.35 ± 0.16	0.59 ± 0.10	0.005
Insulin (mg/dl)	15.6 ± 4.1	53.9 ± 8.1	<0.0001
Glucose (mg/dl)	95.87 ± 9.6	122.37 ± 23.4	0.01
HOMA-IR	3.7 ± 1.04	16.2 ± 3.9	<0.0001
GGT (U/l)	93.14 ± 97.2	76.1 ± 81.08	0.7
GOT (U/l)	32.2 ± 34.02	26.7 ± 12.2	0.6
GPT (U/l)	73 28 ± 82 9	54.37 ± 15.2	0.5
Uric acid (mg/dl)	6.01 ± 1.1	6.17 ± 1.4	0.8
Adiponectin (ug/ml)	11.87 ± 4.02	7.2 ± 2.3	0.02
Leptin (ng/ml)	147.8 ± 105.1	136.6 ± 56.3	0.7

### Anthropometric measurements

The BMI was calculated as weight (in kilograms) divided by height (in meters) squared. The subjects’ waist was measured with a soft tape midway between the lowest rib and the iliac crest. The hip circumference was measured at the widest part of the gluteus region and the waist-to-hip ratio (WHR) calculated.

Blood pressure was measured in the supine position on the right arm after a 10-min rest; a standard sphygmomanometer of appropriate cuff size was used, and the first and fifth phases were recorded. Values used in the analysis are the average of three readings taken at 5-min intervals. Patients were requested to withhold alcohol and caffeine for at least 12 h prior to the insulin-sensitivity test.

### Analytical determinations

Serum glucose levels were measured in duplicate by the glucose oxidase method with a Beckman Glucose Analyzer *2* (*Brea*, *CA*). The coefficient of variation (CV) was 1.9 %. Serum insulin levels were measured in duplicate by monoclonal immunoradiometric assay (IRMA; Medgenix Diagnostics, Fleunes, Belgium). The lowest limit of detection was 4.0 mU/l. The intra-assay CV was 5.2 % at a concentration of 10 mU/l and 3.4 % at 130 mU/l. The inter-assay CVs were 6.9 and 4.5 % at 14 and 89 mU/l, respectively. Frequently sampled intravenous glucose tolerance test with minimal model analysis was performed as previously described [30].

Total serum cholesterol was measured through the reaction of cholesterol esterase/oxidase/peroxidase, using a BM/Hitachi 747. HDL cholesterol was quantified after precipitation with polyethylene glycol at room temperature. Total serum triglycerides were measured through the reaction of glycerol-phosphate-oxidase and peroxidase. Insulin sensitivity was measured using the frequently sampled intravenous glucose tolerance test with minimal model analysis, as previously described [31].

### DNA/RNA Denaturant Gradient Gel Electrophoresis (DGGE)

Total DNA/RNA were extracted from snap-frozen cecum contents using the TriPure reagent according to manufacturer’s protocol, modified by adding a bead (≤ 106 μm diameter)-beating step (6,500 rpm, 3 × 30 s). Then, 200 ng of DNA was amplified by PCR using a *Taq* Polymerase (Sigma Aldrich, St. Louis, MO) and 300 nM DGGE-specific 16S rRNA universal primers (forward primer 5′-CGC CCG GGG CGC GCC CCG GGC GGG GCG GGG GCA CGG GGG GAC TCC TAC GGG AGG CAG CAG T-3′; reverse primer 5′-GTA TTA CCG CGG CTG CTG GCA C-3′), carrying (forward primer only) a GC-enriched region (GC-clamp), generating 233-bp amplicons. The size of the latter was verified by 2 % agarose gel electrophoresis. Then, 80 ng of amplicons was loaded onto 8 % acrylamide gel with a 35–55 % (w/v) urea-denaturant gradient. The gels were run overnight in TAE 1 × , at 60° C. The following day, the gels were stained for 30 min in TAE 1X-*SYBR safe DNA gel staining* and scanned with a Typhoon 9400 instrument (Amersham Biosciences). The band profile was analyzed by the PermutMatrixEN software version 1.9.3.0 [32]. One microgram of total RNA was retrotranscripted for 2 h at 37 °C using the High-Capacity cDNA Reverse Transcription Kit (Applied Biosystems, Villebon-sur-Yvette, France). Ten nanograms of cDNA were amplified using both sense and antisense primers at a concentration of 300 nM.

### DGGE band analysis and Venn’s diagram

DGGE bands were identified and analyzed by using the software ImageQuantTL (GE Healthcare Life Sciences). *Manual lane creation* option was used to draw samples lane. Then, *Edit single lane* option was used to adjust lane width. *Background subtraction* was used to denoise intensity values. Finally, *automatic detection* was applied to identify bands. Venn diagram was constructed based on Oliveros JC website (http://bioinfogp.cnb.csic.es/tools/venny/index.html). No threshold was applied for band intensity, since the background was already subtracted in Venn’s diagrams. Therefore, all bands were analyzed for both DNA (Fig. 2a) and RNA (Fig. 4a).

### Association analysis between DNA bands and insulin resistance

The analysis of the association between DNA bands obtained by DGGE and clinical covariates describing individual characteristics has been performed using multiple statistical techniques whose results have been integrated in order to highlight different relations obtained from diverse approaches. The reason for using this integrative approach is on the one hand because of the small number of individuals (suggesting lack of power) and on the other hand because of the ill-conditioning derived from the number of zeros (suggesting that some approaches may be weaker than usual). Combining different methods allows a more robust approach. Associations appearing in most analyses can be more reliably trusted while those appearing only once can be considered as “potential” relations.

Two types of statistical methods were used to detect bands that could be associated with difference between insulin resistance and insulin sensitivity. By one side, this relation was analyzed through variable HOMA_IR that is clearly associated with this difference. Letting HOMA_IR and insulin sensitivity be the response variables, the relation was modeled through different approaches: quantitative trait analysis to establish correlation between this variable and each band’s values separately, and different regression methods such as Ordinary Least Squares, Principal Components Regression, and Partial Least Squares to find a set of explanatory variables (band) for HOMA_IR. By the other side, multivariate analyses such as Canonical Correspondence Analyses were used to find a set of variables best correlated with the difference between IR and IS accounting for more variables than HOMA_IR.

All analyses were performed using all variables excepted for Quantitative Trait Analysis that was performed on a one-to-one basis and OLS for which a preselection based on significance was done.

A brief description of the statistical methods applied follows below:

- Quantitative Trait Analysis (“QTA” [33]) consists of computing Spearman correlation coefficient between band values and quantitative covariates such as HOMA_IR. Significance is obtained by random permutation testing.
- Linear regression (“OLS”, [34]) has been performed taking HOMA_IR and insulin sensitivity as the dependent variables and bands showing a significant difference between IR and IS patients as explanatory variables. Stepwise regression was used to retain a set of most explanatory bands in the model. The variables were filtered for OLS to have as many variables as individuals.
- Principal Components Regression (“PCR”, [34]) was done by first performing a Principal Components Analysis on all bands, which yield a new set of independent “eigen-bands”, then performing a linear regression, including stepwise variable selection and finally reverting the model to the original scale yielding a coefficient for each band which was used to select bands positively or negatively associated with the response variable.
- Partial least squares (“PLS”, [35]) can be seen as a technique related to Principal Components Regression but which fits a linear regression model by projecting the predicted variables and the observable variables to a new space where the relation between the variables can be better visualized.
- Canonical Correlation Analysis (“CCA”, [36]) is a multivariate method to find correlation between sets of variables. It was performed between a subset of bands obtained by clustering and selecting one canonical representative per cluster and a subset of quantitative clinical variables. An extra regularization step was applied to account for the problem of having more bands than individuals.
- Canonical Correspondence Analysis (“CANOCO”, [37]) is a method developed in Ecology that builds on Correspondence analysis, a multivariate method applicable to analyze cross-tables such as those formed by bands and patients to allow the incorporation of extra explanatory variables in the analysis (here clinical variables).

## References

[1] Ruchat SM, Elks CE, Loos RJ, Vohl MC, Weisnagel SJ (2009). Association between insulin secretion, insulin sensitivity and type 2 diabetes susceptibility variants identified in genome-wide association studies. Acta Diabetol.

[2] Hill JO (2006). Understanding and addressing the epidemic of obesity: an energy balance perspective. Endocr Rev.

[3] Nicholson JK, Holmes E, Wilson ID (2005). Gut microorganisms, mammalian metabolism and personalized health care. Nat Rev Microbiol.

[4] Eckburg PB, Bik EM, Bernstein CN, Purdom E, Dethlefsen L (2005). Diversity of the human intestinal microbial flora. Science.

[5] Ley RE, Turnbaugh PJ, Klein S, Gordon JI (2006). Microbial ecology: human gut microbes associated with obesity. Nature.

[6] Ley RE, Backhed F, Turnbaugh P, Lozupone CA, Knight RD (2005). Obesity alters gut microbial ecology. Proc Natl Acad Sci U S A.

[7] Hotamisligil GS (2006). Inflammation and metabolic disorders. Nature.

[8] Shoelson SE, Lee J, Goldfine AB (2006). Inflammation and insulin resistance. J Clin Invest.

[9] Cani PD, Amar J, Iglesias MA, Poggi M, Knauf C (2007). Metabolic endotoxemia initiates obesity and insulin resistance. Diabetes.

[10] Backhed F, Ding H, Wang T, Hooper LV, Koh GY (2004). The gut microbiota as an environmental factor that regulates fat storage. Proc Natl Acad Sci U S A.

[11] Velagapudi VR, Hezaveh R, Reigstad CS, Gopalacharyulu P, Yetukuri L (2010). The gut microbiota modulates host energy and lipid metabolism in mice. J Lipid Res.

[12] Backhed F, Manchester JK, Semenkovich CF, Gordon JI (2007). Mechanisms underlying the resistance to diet-induced obesity in germ-free mice. Proc Natl Acad Sci U S A.

[13] Samuel BS, Shaito A, Motoike T, Rey FE, Backhed F (2008). Effects of the gut microbiota on host adiposity are modulated by the short-chain fatty-acid binding G protein-coupled receptor, Gpr41. Proc Natl Acad Sci U S A.

[14] Gebbers JO, Laissue JA (2004). Bacterial translocation in the normal human appendix parallels the development of the local immune system. Ann N Y Acad Sci.

[15] Smith HF, Fisher RE, Everett ML, Thomas AD, Bollinger RR (2009). Comparative anatomy and phylogenetic distribution of the mammalian cecal appendix. J Evol Biol.

[16] Turnbaugh PJ, Hamady M, Yatsunenko T, Cantarel BL, Duncan A (2009). A core gut microbiome in obese and lean twins. Nature.

[17] Turnbaugh PJ, Ley RE, Hamady M, Fraser-Liggett CM, Knight R (2007). The human microbiome project. Nature.

[18] Serino M, Chabo C, Burcelin R (2012) Intestinal MicrobiOMICS to define health and disease in human and mice. Curr Pharm Biotechnol 13:746–758 10.2174/138920112799857567 22122483

[19] Amar J, Serino M, Lange C, Chabo C, Iacovoni J et al. (2011) Involvement of tissue bacteria in the onset of diabetes in humans: evidence for a concept. Diabetologia 54:3055–3061 10.1007/s00125-011-2329-8 21976140

[20] Backhed F, Ley RE, Sonnenburg JL, Peterson DA, Gordon JI (2005). Host-bacterial mutualism in the human intestine. Science.

[21] Burcelin R, Luche E, Serino M, Amar J (2009). The gut microbiota ecology: a new opportunity for the treatment of metabolic diseases?. Front Biosci.

[22] Serino M, Luche E, Chabo C, Amar J, Burcelin R (2009). Intestinal microflora and metabolic diseases. Diabetes Metab.

[23] Vrieze A, Holleman F, Zoetendal EG, de Vos WM, Hoekstra JB (2010). The environment within: how gut microbiota may influence metabolism and body composition. Diabetologia.

[24] Nicholson JK, Wilson ID, Lindon JC (2011). Pharmacometabonomics as an effector for personalized medicine. Pharmacogenomics.

[25] Serino M, Luche E, Gres S, Baylac A, Berge M et al. (2012) Metabolic adaptation to a high-fat diet is associated with a change in the gut microbiota. Gut 61:543–553 10.1136/gutjnl-2011-301012 PMC3292714 22110050

[26] Amar J, Burcelin R, Ruidavets JB, Cani PD, Fauvel J (2008). Energy intake is associated with endotoxemia in apparently healthy men. Am J Clin Nutr.

[27] Fernandez-Real JM, Perez del Pulgar S, Luche E, Moreno-Navarrete JM, Waget A (2011). CD14 modulates inflammation-driven insulin resistance. Diabetes.

[28] Moreno-Navarrete JM, Fernandez-Real JM (2011). Antimicrobial-sensing proteins in obesity and type 2 diabetes: the buffering efficiency hypothesis. Diabetes Care.

[29] Fernandez-Real JM, Pickup JC (2011) Innate immunity, insulin resistance and type 2 diabetes. Diabetologia 55:273–278 10.1007/s00125-011-2387-y 22124608

[30] Gubern C, Lopez-Bermejo A, Biarnes J, Vendrell J, Ricart W (2006). Natural antibiotics and insulin sensitivity: the role of bactericidal/permeability-increasing protein. Diabetes.

[31] Fernandez-Real JM, Broch M, Ricart W, Casamitjana R, Gutierrez C (1998). Plasma levels of the soluble fraction of tumor necrosis factor receptor 2 and insulin resistance. Diabetes.

[32] Caraux G, Pinloche S (2005). PermutMatrix: a graphical environment to arrange gene expression profiles in optimal linear order. Bioinformatics.

[33] Simon RM (2003). Design and analysis of DNA microarray investigations.

[34] Rawlings JO, Pantula SG, Dickey DA (1998). Applied regression analysis: a research tool.

[35] Ericksson L, Johansson E, Kettaneh-Wold N, Wold SL (2001). Multi- and megavariate data analysis principles and applications.

[36] Johnson RA, Wichern DW (2007). Applied multivariate statistical analysis.

[37] Lep J, Milauer P (2003). Multivariate analysis of ecological data using CANOCO.

